# New hemodynamic criteria to separate classical orthostatic hypotension from vasovagal syncope

**DOI:** 10.1002/acn3.51412

**Published:** 2021-06-24

**Authors:** Maryam Ghariq, Fabian I. Kerkhof, Robert H. Reijntjes, Roland D. Thijs, J. Gert van Dijk

**Affiliations:** ^1^ Department of Neurology Leiden University Medical Centre Leiden The Netherlands; ^2^ Stichting Epilepsie Instellingen Nederland (SEIN) Heemstede The Netherlands; ^3^ NIHR University College London Hospitals Biomedical Research Centre London United Kingdom

## Abstract

**Objective:**

To define and evaluate hemodynamic criteria to distinguish between classical orthostatic hypotension (cOH) and vasovagal syncope (VVS) in tilt table testing (TTT).

**Methods:**

Inclusion criteria for VVS were a history of VVS and tilt‐induced syncope defined as a blood pressure (BP) decrease and electroencephalographic changes during syncope with complaint recognition. Criteria for cOH were a history of cOH and a BP decrease meeting published criteria. Clinical diagnoses were established prior to TTT. We assessed (1) whether the decrease of systolic BP accelerated, “convex,” or decelerated, “concave”; (2) the time from head‐up tilt to when BP reached one‐half its maximal decrease; (3) the difference between baseline heart rate (HR) and HR at BP nadir. We calculated the diagnostic yield of optimized thresholds of these features and their combinations.

**Results:**

We included 82 VVS cases (40% men, median age 44 years) and 65 cOH cases (66% men, median age 70 years). BP decrease was concave in cOH in 79% and convex in VVS in 94% (*p* < 0.001). The time to reach half the BP decrease was shorter in cOH (median 34 sec, interquartile range (IQR) 19–98 sec) than in VVS (median 1571 sec, IQR 1381–1775 sec, *p* < 0.001). Mean HR increased by 11 ± 11 bpm in cOH and decreased by 20 ± 19 bpm in VVS (*p* < 0.001). When all three features pointed to VVS, sensitivity for VVS was 82% and specificity was 100%. When all three pointed to cOH, sensitivity for cOH was 71% and specificity was 100%.

**Interpretation:**

These new hemodynamic criteria reliably differentiate cOH from VVS.

## Introduction

Orthostatic intolerance (OI) refers to symptoms when standing upright which are relieved by reclining.^1^ OI is common and the causes may be confused. Notably, classical orthostatic hypotension (cOH) and vasovagal syncope (VVS) are both triggered by orthostatic stress and may both result in syncope. Syncope is the form of transient loss consciousness (TLOC) that is caused by global cerebral hypoperfusion; it is characterized by a rapid onset, short duration, and complete and spontaneous recovery.^1^ Both cOH and VVS are common causes of syncope.^2^, ^3^, ^4^, ^5^, ^6^. The tilt table test (TTT) plays an important role in the work‐up of those with unexplained OI or TLOC.^1^, ^7^, ^8^, ^9^, ^10^

In VVS, syncope during TTT is always accompanied by a decrease of blood pressure (BP) and often also of heart rate (HR). The hemodynamic patterns of VVS are commonly categorized using the VASIS (Vasovagal International Study) criteria.^11^, ^12^ cOH is defined as a sustained drop of systolic BP of at least 20 mmHg and/or a drop in diastolic BP of at least 10 mmHg, within 3 min of standing or tilt‐up of at least 60° on a tilt‐table.^1^, ^13^, ^14^, ^15^, ^16^

The two sets of criteria, for VVS and cOH, were not designed to distinguish between one another. A comparison reveals potential problems. Starting with cOH, the cOH criteria do not provide clues to help exclude VVS or other forms of reflex syncope during TTT, as there is no mention of the behavior of HR. The maximum period of 3 min in which BP must decrease after head‐up tilt will exclude the majority of VVS during TTT, but in a minority of cases, VVS can occur that quickly. The mention of “sustained” low BP for cOH also argues against VVS being mistaken for cOH, but “sustained” is not specified; moreover, BP in cOH can fall so quickly that patients may have to be tilted back before a “sustained” state van be ascertained.^17^ Furthermore, delayed OH (dOH) may pose a problem, as the time until a BP decrease becomes abnormal is then by definition more than 3 min after head‐up tilt, so its latency range overlaps with that of VVS.^18^, ^19^

To continue with VVS, the VASIS criteria for TTT distinguish between forms of VVS and do not exclude cOH or dOH. The VASIS emphasis on cardioinhibition, that is, an HR decrease, should make it unlikely that the cardioinhibitory types 2A and 2B are mistaken for cOH, although this presumes knowledge that HR does not decrease during OH, something not mentioned in the cOH criteria. Type 3, the vasodepressor type, and those cases of type 1 (“Mixed”) in whom HR stays well above 40 bpm, may well be mistaken for cOH.

We, therefore, formulated and tested three additional criteria to help differentiate cOH and VVS during TTT: first, the shape of the BP decrease, that is, accelerating in VVS, decelerating in cOH; second, the latency of the BP decrease: long in VVS, short in cOH; third, HR change at BP nadir: up in cOH, down in VVS, or unchanged in both.

## Methods

We based the selection of the three possible additional features to help differentiate cOH and VVS on clinical and TTT experience. The first is the “shape of the BP decrease”: the BP decrease tends to accelerate in VVS but decelerates in cOH. Second, the BP nadir tends to occur later after head‐up tilt in VVS than in cOH. Third, HR either goes up or stays stable in cOH while BP decreases, while in VVS it goes down or stays stable.

### TTT protocol

Routine TTT comprised continuous blood pressure (BP) measurement with either a Finometer^®^ (Finapres Medical Systems, Amsterdam, the Netherlands) or a Nexfin® (BMEye, The Hague, the Netherlands), a one channel electrocardiogram (ECG), and always video and electroencephalography (EEG).^20^ A neurology resident and a technician had continuous access to all signals during the test.

The Leiden TTT protocol for VVS has been described before.^18^, ^21^ In short, we used a modified Italian protocol with 10 min of supine rest, a passive phase of 20 min of 60–70° of head‐up tilt, followed by the sublingual administration of 400 *µ*g of nitroglycerin (NTG) and another 20 min of head‐up tilt.

The Leiden TTT protocol for suspected cOH consisted of 10 min of supine rest and 20 min of 60–70° of head‐up tilt without the use of NTG. The test was terminated earlier when syncope occurred or when patients had complaints of the OH.

### Inclusion criteria

Inclusion for both cOH and VVS rested on the clinical diagnosis, reached before TTT, as well as on TTT findings. We selected patients with VVS or cOH who had undergone a TTT between January 1st, 2006 and December 31st, 2016 in the Leiden University Medical Centre.

The clinical diagnosis VVS was made by neurologists familiar with VVS (JGvD and RDT); the cOH diagnosis could also be made by movement disorder neurologists.

Clinical exclusion criteria were an age <16 years and missing clinical data. Dutch law did not require individual informed consent for the publication of anonymous data gathered exclusively in the context of patient care, as was the case here, in the period in which the data were gathered.

The TTT criterion for VVS was defined, using a triad of clinical symptoms or signs, EEG changes, and a decrease in BP as in previous studies.^21^, ^22^ Syncope could occur with or without NTG provocation.^18^ In both cases, the time was determined from the start of tilt table verticalization. All patients had to recognize complaints during TTT. For this study, we excluded presyncope by demanding EEG slowing during the clinical loss of consciousness. The start of the loss of consciousness was defined as the moment where purposeful movements and verbal responses disappeared or facial expression became vacant. We tilted patients back when syncope occurred when the allotted time had passed, or when EEG slowing or asystole occurred. When video or EEG were poor in quality or absent, cases were excluded. Patients with an additional cause of transient loss of consciousness during TTT (e.g., psychogenic pseudosyncope) were also excluded.

The TTT criterion for cOH conformed to consensus TTT criteria.^14^ If the patient‐reported symptoms during TTT, we stipulated that these had to be recognized as identical to the referral reason for inclusion. Further workup in cases of cOH depended on the etiological likelihood and could involve additional autonomic testing (Ewing battery, active standing), brain MRI, MIBG‐scanning of the heart, and other procedures. These procedures are not discussed here.

### Measurements

#### Shape of BP decrease

**Figure 1:**
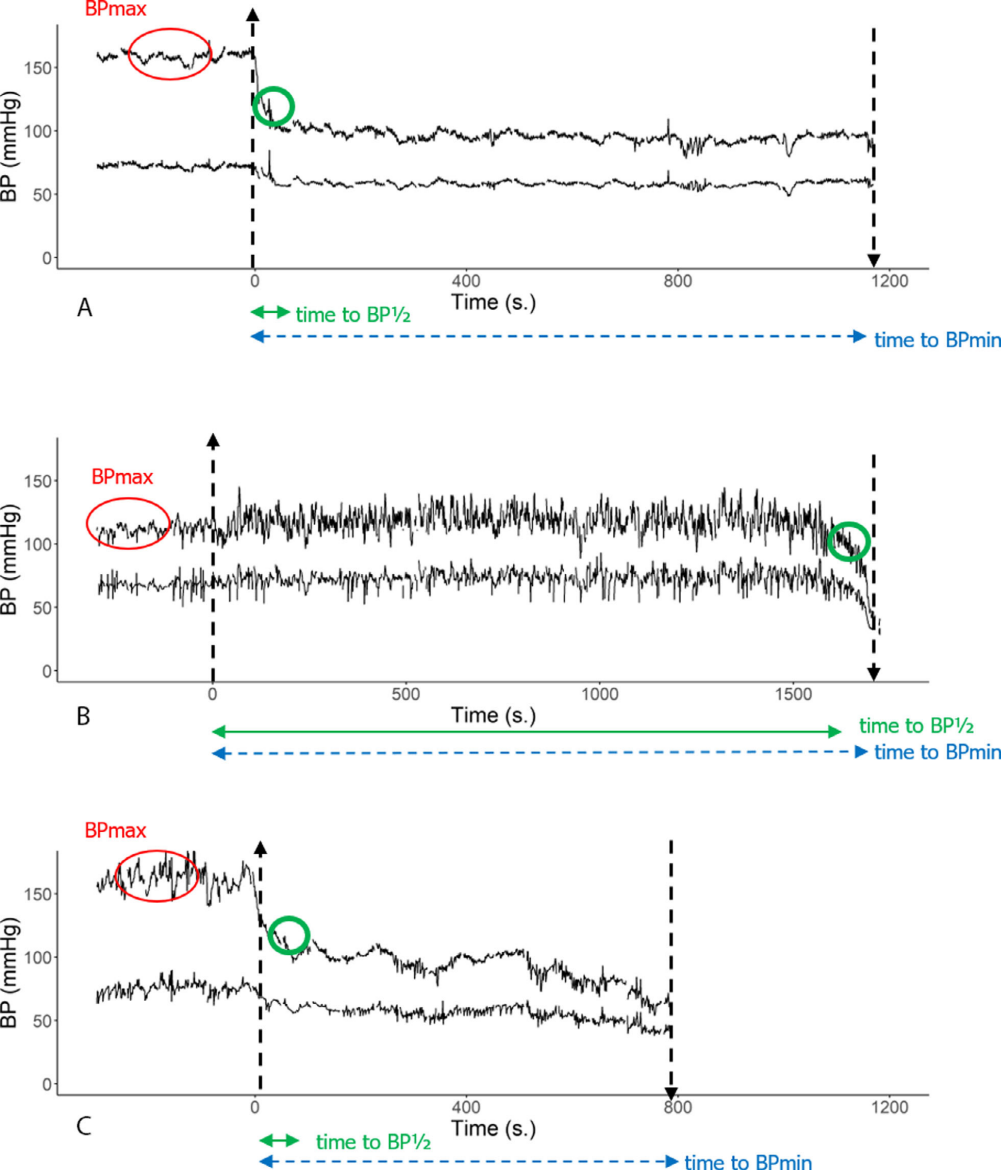
Examples of different blood pressure shapes. The blood pressure (BP) curve in panel (A) shows a decelerating “concave” BP decrease. The graph in panel (B) shows an accelerating “convex” decrease just before nadir. Patterns with both decelerations and accelerations were classified as “unclassifiable,” of which panel (C) shows an example: a decelerating BP drop is followed first by a plateau and then by an accelerating BP drop.

We used a consensus procedure among a panel of four examiners (JGvD, RDT, FK, MG) to define the shape of the BP decrease toward the minimum BP as “convex,” “concave,” or “unclassifiable.” For each case, we presented a graph of the smoothed systolic BP to emphasize the overall shape of the BP decrease. Having tried several smoothing periods, we used a 181 sec window (from 90 sec before to 90 sec after each point). For the first and last 90 sec of the TTT, the smoothing window was adjusted to fit available data. As a result, smoothing was less pronounced in the periods where changes are quickest and most relevant (early for cOH and late for VVS). We noted a “convex shape” when the BP decline accelerated just before syncope, (Fig. 1), and a “concave shape” when the rate of BP decline decreased before reaching the BP nadir. BP patterns with both deceleration and acceleration were labeled “unclassifiable.” If no consensus was reached this was noted, but the case was excluded from further analysis.

#### Latency of BP decrease

We calculated BP before tilt‐up, labelled “BPmax,” as the average BP of the period from 300 to 120 sec before tilting up. The minimum BP (“BPmin”) in VVS occurred at syncope, and in cOH, it usually occurred just before patients were tilted back. We measured BPmin in that period, now using smoothed BP with a smaller window of 15 sec before to 15 sec after each point. Smoothing was performed to reduce the effect of fluctuations and outliers. We calculated the time to reach BPmin from head‐up tilt, as well as the difference between BPmax and BPmin. To quantify the latency of the BP decrease, we did not simply use the time to reach BPmin, as in subjects with cOH BP can remain stably low for a long time or continue to decrease very slowly as long as patients are kept upright. Instead, we used the time from tilt up to reach half the difference between BPmax and BPmin (“BP½ time”), a parameter that does not suffer from these defects and should also be sensitive to the accelerating or decelerating nature of the BP decrease. For the calculations of diagnostic yield (see below), we expressed BP½ time as a percentage of the time to reach BPmin.

#### HR change at BPmin

We calculated that mean HR for the same time slots was also used to define BPmax and BPmin. The difference between HR at BPmax and BPmin was calculated.

### Statistical analysis

Results were presented as mean ± SD for continuous variables with a normal distribution and as medians with interquartile range for data that were not normally distributed. We used the Mann–Whitney *U* test and Student's *t*‐test where applicable.

The first analysis step assessed whether the three features differed between the VVS and cOH groups. We used the Chi‐square test for the BP shape, and analyzed the latency of BP decrease and HR change with the Mann–Whitney *U* test.

The second step involved the diagnostic value of the three features to distinguish between cOH and VVS. Arguments for VVS were a convex shape, a short latency, and a decrease of HR, and for cOH they were a concave BP shape, a long latency, and no HR decrease. This required a dichotomization of the three features, already described for the BP shape. To dichotomize BP latency and HR change, we used a receiver operating curve (ROC) to calculate the percentage that discriminated best between VVS and cOH.

After dichotomization, we calculated sensitivity, specificity, the positive and negative likelihood ratios for each of the three features, separately for VVS and for cOH. We also calculated the diagnostic yield for all combinations of two or three features. All analyses were performed using IBM SPSS Statistics version 23 (SPSS Inc, Chicago, IL). Statistical significance was set at *p* < 0.05.

## Results

### Subject characteristics

**Figure 2:**
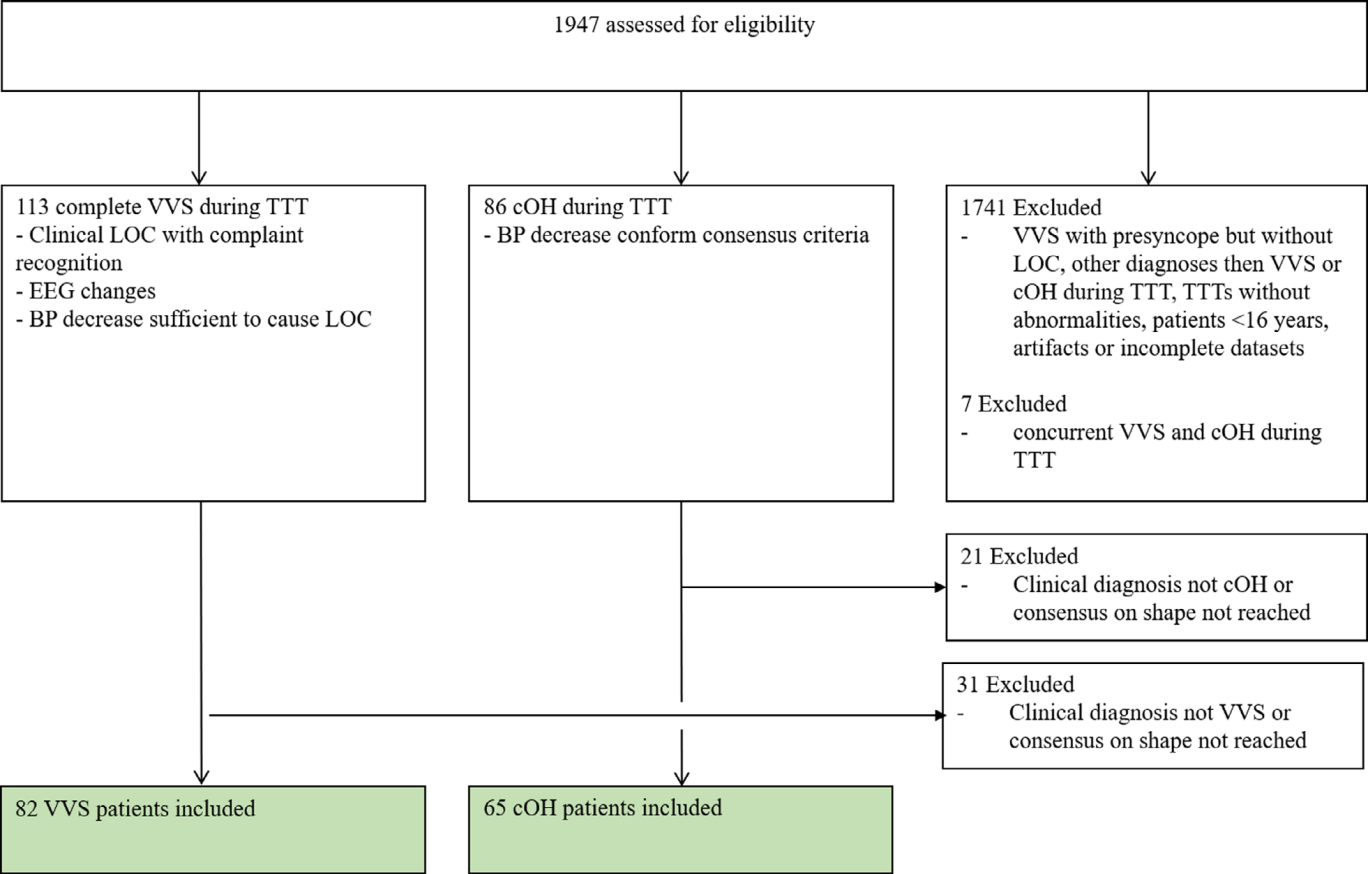
Flow chart of patient selection.

Between January 1st, 2006 and December 31st, 2016, 1947 TTTs were performed for transient loss of consciousness or orthostatic intolerance. The application of inclusion and exclusion criteria left 147 patients (Fig. 2): 65 with cOH (43 men, 66%) and 82 with VVS (33 men, 40%). Patients with VVS were younger (median age 44, range 16–77 years) than cOH patients (median age 70, range 37–89 years; *p* < 0.001). (Table 1) In the cOH group, 83% had primary autonomic failure (Parkinson’s disease, multiple system atrophy, pure autonomic failure), 14% secondary autonomic failure (polyneuropathy, diabetes, amyloidosis), and 3% drug‐induced OH.

**Table 1:** Patient characteristics and hemodynamic features of vasovagal syncope and classical orthostatic hypotension during tilt table testing.

	VVS (*n* = 82)	cOH (*n* = 65)	
Sex	33 men (40%)	43 men (66%)	
Age, years (median, range)	44 (16–77)	70 (37–89)	*p* < 0.001 (MWU)

Shape of BP curve	VVS	cOH	
Convex	77	1	*p* < 0.001 (Chi square)
Concave	0	51	
Unclassifiable	5	13	

Latency of BP decrease	VVS	cOH	
Time to BP½ in s (median, IQR)	1571 (1381–1775)	34 (19–98)	*p* < 0.001 (MWU)
Time to BPmin s (median, IQR)	1614 (1406–1841)	778 (328–1040)	*p* < 0.001 (MWU)

HR change at BPmin^1^	VVS	cOH	
Mean change in bpm (mean, ±SD)	−19.5 ± 18.5	10.8 ± 11.3	*p* < 0.001 (*t*‐test)

Abbreviations: BP, blood pressure; bpm, beats per minute; cOH, classical orthostatic hypotension; HR, heart rate; IQR, interquartile range; MWU, Mann–Whitney *U* test; s, seconds; SD, standard deviation; VVS, vasovagal syncope.

^1^ Average HR supine minus HR at BP nadir during tilt table testing.

### Group differences

#### Shape of BP decrease

In the cOH group, 51 patients (78.5%) had a concave BP shape, one a convex shape, and the shape was unclassifiable in 13 patients. In the VVS group, 77 patients (93.9%) had a convex shape, none a concave shape, and the shape was unclassifiable in five (6.1%) (*p* < 0.001) (Table 1).

#### Latency of BP decrease

**Figure 3:**
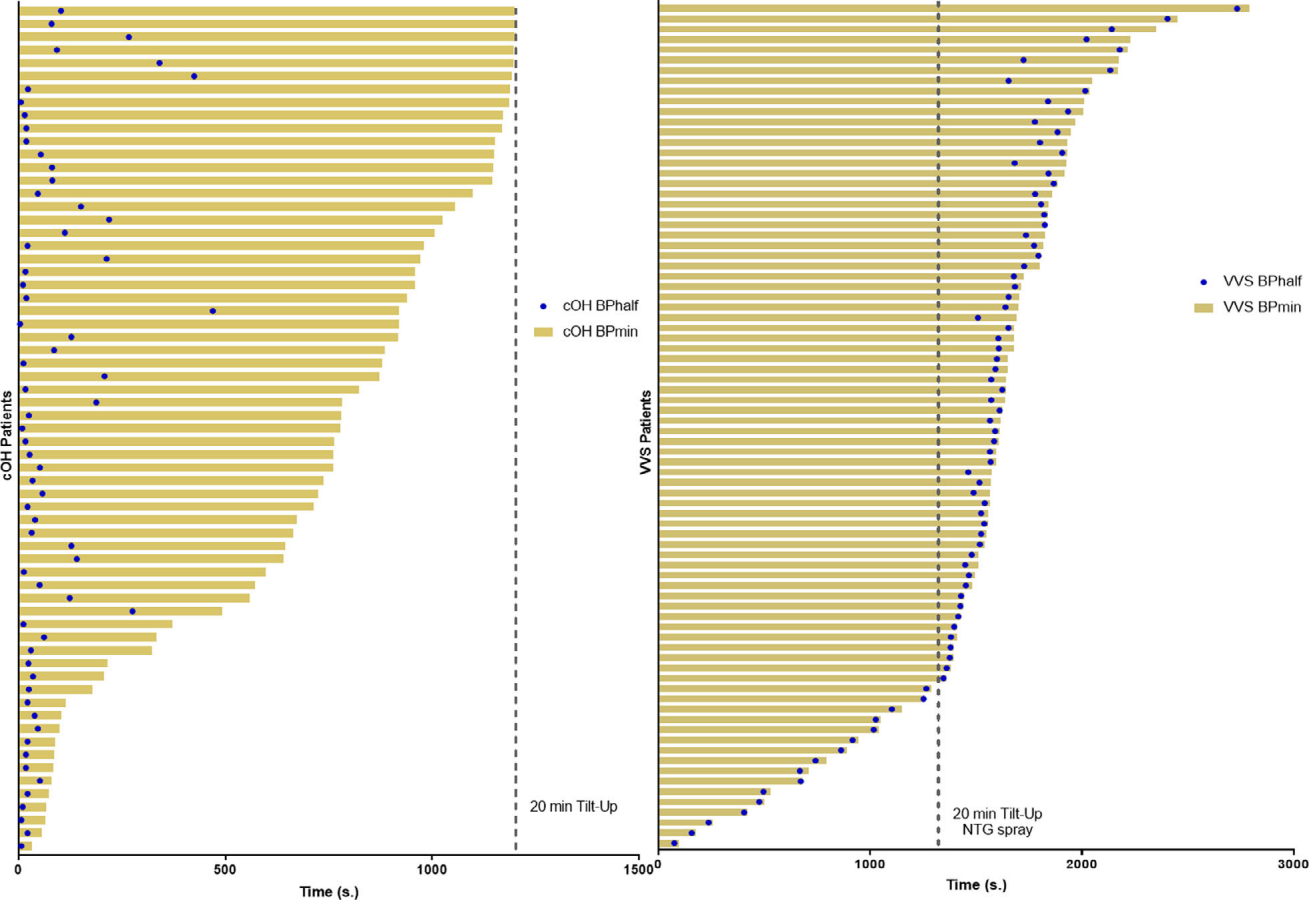
Relative time to one‐half the blood pressure decrease compared to the time to lowest blood pressure during tilt‐up. Each bar represents one patient. The blue dots represent the time in seconds when one‐half of the maximal blood pressure (BP) decrease was reached during tilt‐up. The length of the yellow bar indicates the time until BPmin in seconds. In cOH patients, half of the BP decrease was reached directly after tilt‐up, whereas patients with VVS reached half of BP decrease just before tilt‐back.

The time to BPmin was much shorter in the cOH group (median 778, range 328–1040 sec) than in the VVS group (median 1614, range 1406–1841 sec, *p* < 0.001). The time to BP½ was also much shorter in the cOH group (median 34, range 19–98 sec) than in the VVS group (median 1571, range 1381–1775 sec, *p* < 0.001) (Table 1, Fig. 3).

#### HR change at BPmin

**Figure 4:**
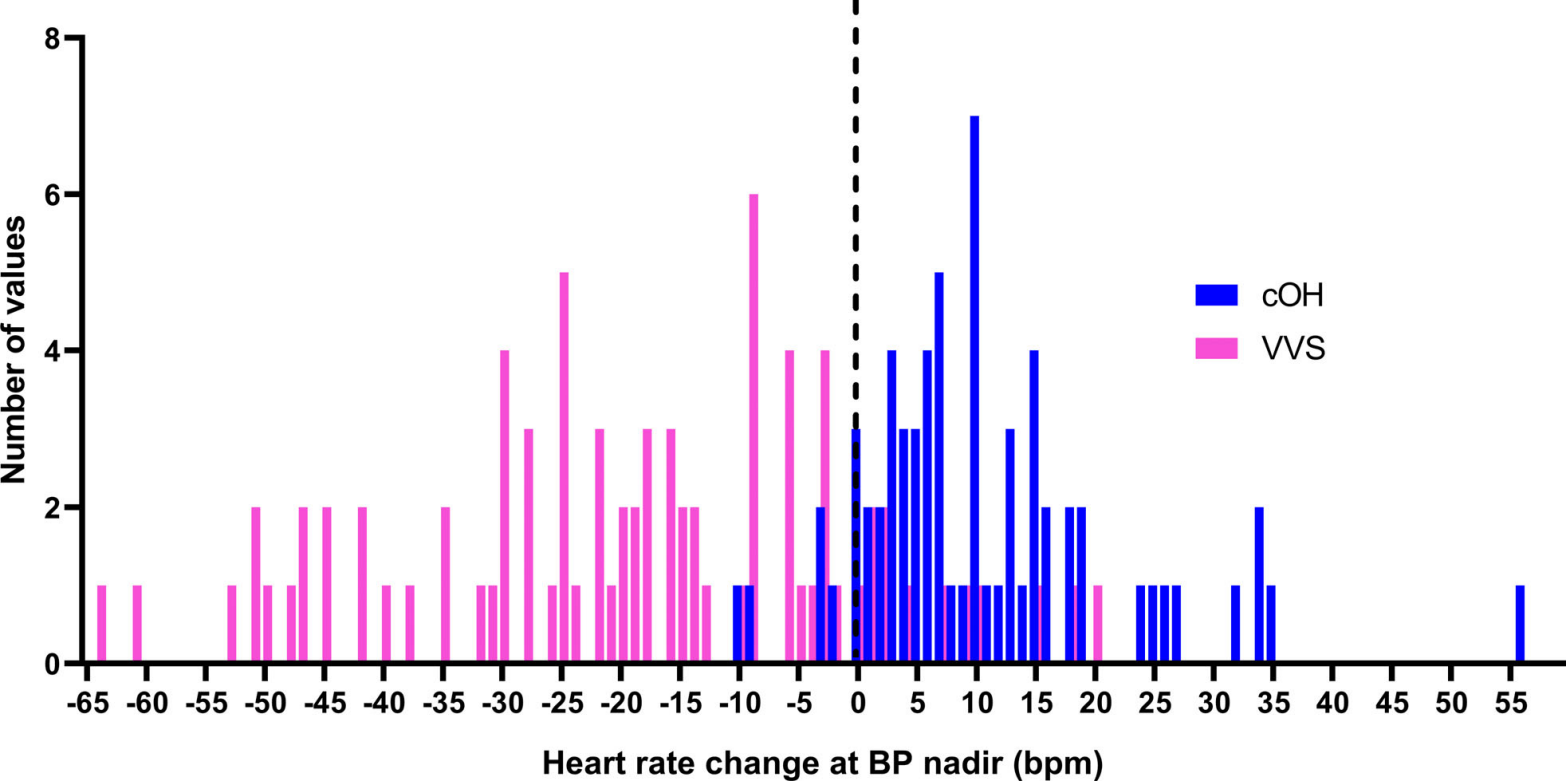
Average heart rate (HR) supine minus HR at blood pressure nadir during tilt table testing. The dotted line represents the value (−0.03 beats per minute) with the highest discriminatory rate between vasovagal syncope and classical orthostatic hypotension.

The mean HR decrease was 20 ± 19 bpm in the VVS group, whereas in the cOH group, HR increased by 11 ± 11 bpm (*p* < 0.001). (Table 1, Fig. 4).

### Diagnostic yield

A BP latency percentage of 72% yielded the best combination of sensitivity (100%) and specificity (100%) to distinguish between cOH and VVS (area under the curve (AUC) = 1.00, 95% confidence interval (CI) = 1.00–1.00, *p* < 0.001). For the HR change at BP nadir, an HR decrease of 0.03 bpm provided the optimal ability to discriminate between groups, with a sensitivity (87%) and specificity (91%), between VVS and cOH (AUC = 0.93, 95% CI 0.89–0.98, *p* < 0.001). (Tables 2 and 3).

**Table 2:** The diagnostic value of the three features for vasovagal syncope.

Shape of BP curve	VVS	cOH	Total
Convex	77	1	78
Concave or unclassifiable	5	64	69
	Sensitivity for VVS: 94%	Specificity for VVS: 98%	147
Latency of BP½ decrease			
Half of BP decrease latency under 72%	0	65	65
Half of BP decrease latency over 72%	82	0	82
	Sensitivity for VVS: 100%	Specificity for VVS: 100%	147
HR change at BP nadir			
HR decreases more than 0.03 bpm	71	6	77
HR increases or is unchanged	11	59	70
	Sensitivity for VVS: 87%	Specificity for VVS: 91%	147

A BP½ latency percentage of 72% yielded the best combined sensitivity (100%) and specificity (100%) to distinguish VVS from cOH (area under the curve (AUC) = 1.00, 95% confidence interval (CI) = 1.00–1.00, *p* < 0.001). For the HR change at BP nadir, an HR decrease of 0.03 bpm at BPmin provided the optimal ability to discriminate between groups, with a sensitivity (87%) and specificity (91%) for VVS (AUC = 0.93, 95% CI 0.89–0.98, *p* < 0.001).

**Table 3:** The diagnostic value of the three features for classical orthostatic hypotension.

Shape of BP curve	cOH	VVS	Total
Concave	51	0	51
Convex or unclassifiable	14	82	96
	Sensitivity for cOH: 78%	Specificity for cOH: 100%	147
Latency of BP½ decrease			
Half of BP decrease latency under 72%	65	0	65
Half of BP decrease latency over 72%	0	82	82
	Sensitivity for cOH: 100%	Specificity for cOH: 100%	
HR change at BPmin			
HR decreases more than 0.03 bpm	6	71	77
HR increases or is unchanged	59	11	70
	Sensitivity for COH: 92%	Specificity for cOH: 87%	147

A BP½ latency percentage of 72% yielded the best combined sensitivity (100%) and specificity (100%) to distinguish cOH from VVS (area under the curve (AUC) = 1.00, 95% confidence interval (CI) = 1.00–1.00, *p* < 0.001). For the HR change at BP nadir, an HR decrease of 0.03 bpm at BPmin provided the optimal ability to discriminate between groups, with a sensitivity (87%) and specificity (91%) for cOH (AUC = 0.93, 95% CI 0.89–0.98, *p* < 0.001).

Table 4 shows the diagnostic yield for all features and combinations. For example, the sensitivity of 94% for BP shape for VVS reflects the presence of a convex BP decrease and the specificity of 98% in its absence. All three features showed high sensitivity and specificity. Combinations of these features generally showed lower sensitivity than the separate features, but with a specificity of 100%.

**Table 4:** Diagnostic value of the three features and these features together.

	VVS				cOH			
	Sensitivity	Specificity	LR+	LR−	Sensitivity	Specificity	LR+	LR−
(A) Shape of BP curve (concave for cOH and convex for VVS)	94%	98%	64	0.061	78%	100%	–	0.22
(B) Latency of BP½ decrease	100%	100%	–	0	100%	100%	–	0
(C) HR change at BPmin	87%	91%	11	0.14	91%	87%	7	0.09
Combinations								
(A) AND (B) AND (C)	82%	100%	–	0.18	71%	100%	–	0.29
(A) + (B)	94%	100%	–	0.06	78%	100%	–	0.22
(A) + (C)	82%	100%	–	0.18	71%	100%	–	0.29
(B) + (C)	87%	100%	–	0.13	91%	100%	–	0.09

Abbreviations: BP, blood pressure; cOH, classical orthostatic hypotension; HR, heart rate; LR−, negative likelihood ratio; LR+, positive likelihood ratio; VVS, vasovagal syncope.

## Discussion

### Main findings

The main finding of our study was that three new hemodynamic features distinguished very well between cOH and VVS. The feature with highest sensitivity and specificity was the latency to reach half the overall BP decrease: this was short for cOH and much longer for VVS. The second‐best feature was the concave or convex shape of the BP decrease, and the HR response to low BP performed least of the three features, but still well.

### Discussion of each feature and of the combinations

The shape classification depended on a visual estimate, but the high consensus rate in the present study suggests that shape classification is robust. Admittedly, cases with less obvious BP patterns may not lend themselves to a dichotic classification, but this was the case in only 13 percent of all patients in this cohort.

Dividing the latency to reach half the maximal BP decrease resulted in a perfect diagnostic yield to differentiating cOH from VVS. One may argue that our protocol for cOH, with a 20‐min period after head‐up tilt, may have influenced the results, as a longer period may have allowed BP to decrease more. However, all cOH cases fulfilled the criterion that systolic BP decreased by at least 20 mmHg within 3 min of tilt‐up, even though BP could decrease further during the remaining 17 min of head‐up tilt. The VVS data show that only 2% of patients developed syncope within 3 min after head‐up tilt. Thus, the time to reach half of BP decrease did not depend strongly on the duration of head‐up tilt.

HR change had the overall lowest diagnostic yield, but this is relative: the positive likelihood ratio (LR+) values of 11 and 7 were in fact impressively high. The reason that this feature had the lowest yield probably lies in the pathophysiology of both disorders: in vasovagal syncope an elevated parasympathetic activity or an impaired sympathetic outflow can cause bradycardia, but no HR change is also possible.^11^, ^12^, ^23^, ^24^ In cOH, HR does not decrease.^25^ HR should normally increase to compensate for a BP decrease in cOH, but this HR increase is less pronounced or absent in neurogenic cOH.^26^ Hence, the absence of an HR response occurs in both cOH and VVS. Of note, the HR criterion proved valuable even in the context of our cOH study population, with predominantly neurogenic cOH and limited HR reactivity.

Various combinations of the three features proved to have perfect specificity as well as reasonable sensitivity for cOH, and very good sensitivity for VVS. Hence, we suggest to base the overall categorization of the results as cOH or VVS on all three features. In daily practice, not all three may be necessary, but experience suggests that having all three features are advantageous when judging complex cases and conditions. In clinical practice, we found that it is not usually necessary to calculate the parameters described in this study; a quick visual analysis of the HR and BP patterns suffices in most cases.

### Causes and consequences

The abovementioned hemodynamic differences must point to as yet unidentified fundamental differences in the pathophysiology between cOH and VVS. In short, arterial BP is the product of total peripheral resistance (TPR), HR, and stroke volume (SV).^18^ On standing, 0.5 to 1 L of blood shifts from the upper to the lower body, leading to a decrease in SV.^27^ In normal circumstances, this decrease is counteracted by increases of HR or TPR. In cOH, this compensation fails; whether this depends primarily on the deficient regulation of SV, HR or TPR may well depend on the cause of cOH.^28^, ^29^, ^30^ Further studies are needed to explain the mechanism of the immediate, fast, and decelerating BP decrease in cOH.

Orthostatic VVS starts with slow venous pooling causing a decrease in SV.^18^ A recent study attributed to the decrease of BP at syncope in tilt‐induced VVS in about equal measure to a slow decrease of SV and a later quick decrease of HR.^18^ The slow initial decrease of SV in VVS probably explains the long latency toward a significant BP decrease, but does not yet explain why the BP decrease accelerates. Again, detailed hemodynamic studies will be needed to understand the detailed mechanisms.

Meanwhile, our results may improve the diagnostic accuracy of the TTT and avoid misdiagnosis of cOH and VVS. As previously noted, the present study represents the first step to assess the efficacy of the three new features, which is why we confined the study to cases with a firm clinical diagnosis of cOH or VVS. We have not formally tested how the criteria perform if the initial evaluation fails to provide a likely diagnosis, but we expect that a clear TTT pattern will then help establish the diagnosis. The three “Leiden criteria” described in this paper are not meant to replace the VASIS criteria for VVS nor the consensus statement for cOH, but may be used additionally to help discriminate between cOH and VVS.

### Limitations

The study concerns a large number of patients in whom the diagnosis was based on separately established clinical and TTT evidence. The TTT protocol with continuous BP measurement and video‐EEG allowed detailed data scrutiny.^20^, ^21^ However, the study concerns a single‐center study with a possible bias induced through patient selection or protocol choices. The visual approach of classifying the BP decrease might be subjective. However, four investigators, blinded for the diagnosis, independently assessed the BP shape in a consensus meeting to limit any subjectivity.

In clinical practice, the presence of emotional triggers for VVS should exclude any confusion with cOH, but in such cases, there usually is no need to perform TTT.^1^, ^10^ Even so, it is unknown whether spontaneous emotional VVS has the same hemodynamic features as orthostatic VVS we described here. This should not pose any problems, as those with emotional VVS are susceptible to TTT.^31^, ^32^ The hemodynamic pattern that such patients then show is that of orthostatic VVS.

Those with TTT experience may feel that the features described in this study conformed to experience; we have, in fact, mentioned the importance of the BP shape previously.^18^, ^21^, ^33^ However, these features have neither been specifically defined nor quantified previously.

We did not compare TTT results with other tests that can be applied in cOH, such as the Valsalva test or the active stand test.^10^ The active stand test usually lasts shorter than a TTT, and so is less likely to evoke VVS, but this is not impossible. We expect that the overall hemodynamic features of VVS and cOH during active stand will be similar to those during TTT.

We did not yet investigate differences between various causes of cOH, such as hypovolemia, drug‐induced cOH, and neurogenic cOH, but it is important to note that the majority of our cOH cases suffered from neurogenic causes. Previous work indicated that the heart rate profiles may differ between cOH groups.^26^ We have also not studied delayed OH (dOH) and do not extrapolate our results to dOH,^19^ that might concern a mixture of the processes involved in OH and VVS.^34^

## Conclusion

Three characteristic hemodynamic features help to differentiate between cOH and VVS: the shape of overall BP fall, the latency of BP decrease, and the direction of HR change. These hemodynamic contrasts likely rest on a failure of compensation mechanisms upon standing in cOH, whereas VVS is characterized by initial slow venous pooling followed by subsequent cardioinhibition. These underlying pathophysiological mechanisms require further study.

## Conflict of Interest

M. Ghariq, F.I. Kerkhof, and R.H.A.M. Reijntjes report no disclosures relevant to the manuscript. J.G. van Dijk has received lecture fees from Medtronic. R.D. Thijs has received fees for lectures from Medtronic, UCB, and Novartis. R.D. Thijs has received consultancy fees from Arvelle and Theravarance. R.D. Thijs receives research support from the Dutch National Epilepsy Fund, The Netherlands Organisation for Health Research and Development (ZonMW; 843002707), NUTS Ohra Fund, Medtronic, NewLife Wearables, and the Christelijke Vereniging voor de Verpleging van Lijders aan Epilepsie, The Netherlands.

## Author Contributions

JGvD, MG, and RDT conceptualized the study and designed it. MG, RHR, and FIK did the data acquisition and prepared the first draft of the manuscript. All authors were involved in the interpretation of data, critically reviewed the manuscript, and approved the final version.

## Data Availability

Anonymized data can be made available to qualified investigators on reasonable request.
